# Keratin-A6ACA NPs for gastric ulcer diagnosis and repair

**DOI:** 10.1007/s10856-021-06537-3

**Published:** 2021-06-12

**Authors:** Yi Ding, Run Meng, Haimeng Yin, Zongkun Hou, Changfa Sun, Wenjie Liu, Shilei Hao, Yun Pan, Bochu Wang

**Affiliations:** 1 Key Laboratory of Biorheological Science and Technology, Ministry of Education, College of Bioengineering, Chongqing University, Chongqing, 400030 China; 2 Department of Gastroenterology, Children’s Hospital of Chongqing Medical University, Chongqing, 400014 China

## Abstract

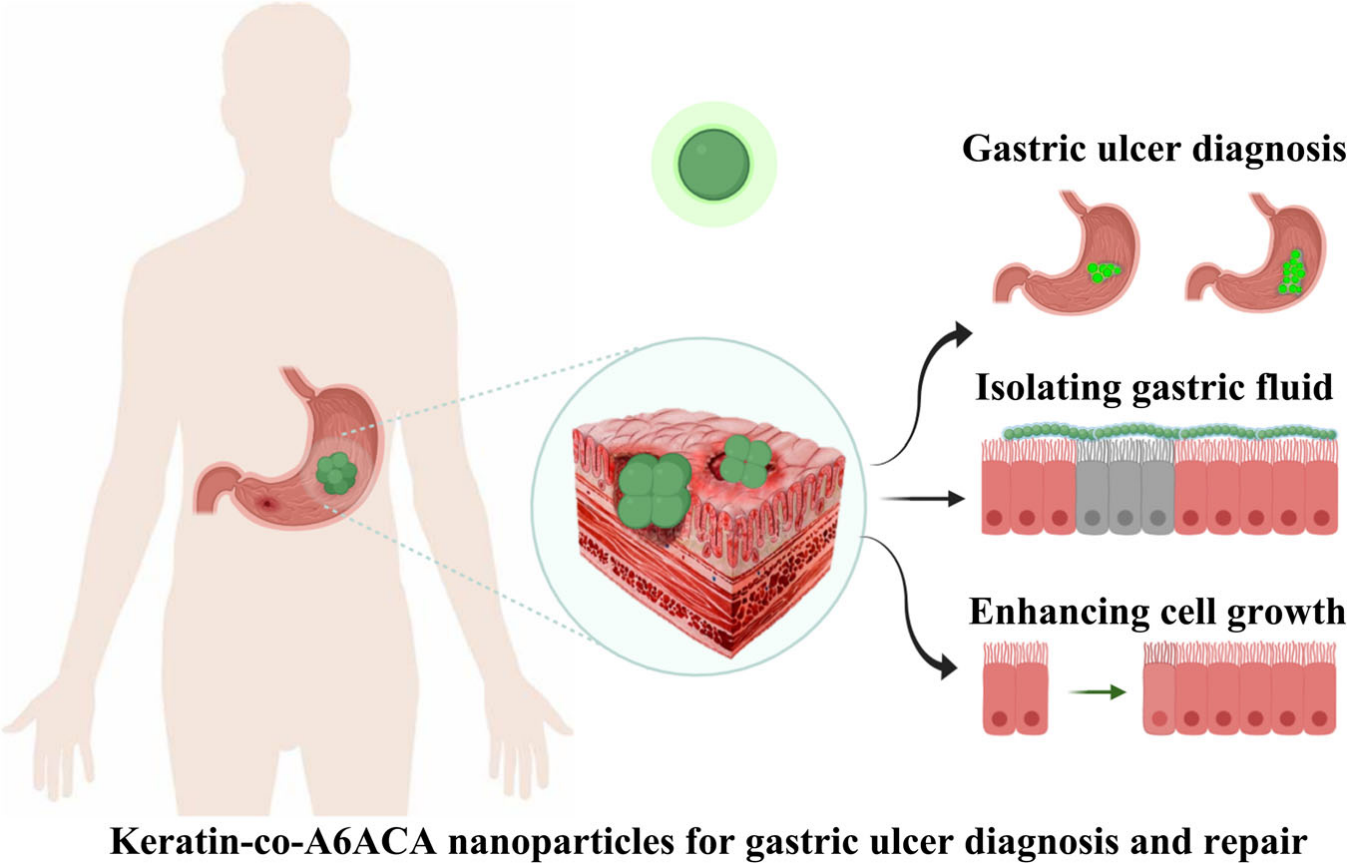

## Introduction

Gastric ulcer is one of the most common diseases affecting global human health [1, 2]. But the effective diagnosis and repair of gastric ulcer remain a challenge in clinic. Endoscopy was frequently used for gastric ulcer diagnosis, which was also used to assist the biomaterial-mediated gastric ulcer therapy [3]. However, the complications of endoscopy have been found [4]. Here, keratin conjugated acryloyl-6-aminocaproic acid nanoparticles (K-co-A6ACA NPs) entrapped with IR783 were fabricated for early diagnosis and treatment of gastric ulcer in mice without endoscopy guidance. Human hair keratin could adhere to the gastric ulcer due to the viscosity increase, and A6ACA was conjugated to keratin to protect IR783 and gastric epithelial cells from gastric juice. The fluorescent signal of IR783-loaded K-co-A6ACA NPs can be clearly observed in the mice stomach, and higher fluorescence intensity can be found in case of larger ulcer formed in mice stomach. While, the fluorescence from IR783 was quenched in acidic condition. Furthermore, the strong ulcer repair properties of K-co-A6ACA NPs were contributed by the isolating effect of A6ACA and ulcer targeting and wound healing effects of keratin.

## Materials and Methods

Keratin was extracted from human hair via a reductive method [5], and the keratin conjugated A6ACA (K-co-A6ACA) was synthesized by EDC/NHS reaction. Briefly, A6ACA (0.3 g) was dissolved into MES buffer (pH 6.0), EDC (0.0575 g) and NHS (0.0115 g) were dissolved into keratin solution (0.6 g). The reaction was performed at room temperature for 24 h (pH 7.5).

The dye-loaded K-co-A6ACA NPs were prepared using O/W/O method. IR783 (5 mg) was dissolved into dichloromethane (150 μL) as inner oil phase, and K-co-A6ACA (300 mg), N, N methylene bisacrylamide (19.96 mg), ammonium persulfate (72.72 mg) and TEMED (51.21 μL) were dissolved into 1.6 ml of NaOH (1 mol/L). The O/W emulsion was prepared by adding the inner oil phase into NaOH, which was then added into the 6 mL of liquid paraffin to form O/W/O emulsion. The NPs were washed three time using deionized water.

The FTIR spectra, particle size, and morphology of NPs were evaluated. All animal experiments were guided by the Animal Ethical and Experimental Committee of the Third Military Medical University, China. IR783-loaded NPs and IR783 were fed into the SPF BABL/C female mice, and the in vitro fluorescence measurements under different pH conditions were performed using an IVIS Lumina XRMS imaging system. In addition, all mice weighting 20–22 g were fed with different amount of absolute ethanol (3 and 6 mL/kg) to induce experimental gastric ulcer with different ulcer areas. Moreover, 6 mg/kg of keratin, A6ACA, NPs, and bismuth potassium citrate (BPC) were selected to treat gastric ulcer, and histological changes of stomach were evaluated. Besides, the levels of TNF-α, IL-6, and IL-1β in serum and the SOD, MDA, and MPO in the gastric tissue were detected. The SPSS 13.0 was used to statistically analyze the experimental results. Tukey test was used to analyze the differences between the means of each experimental group and the control group. The statistically significant level (P) was set to be <0.05.

## Results

**Fig. 1:**
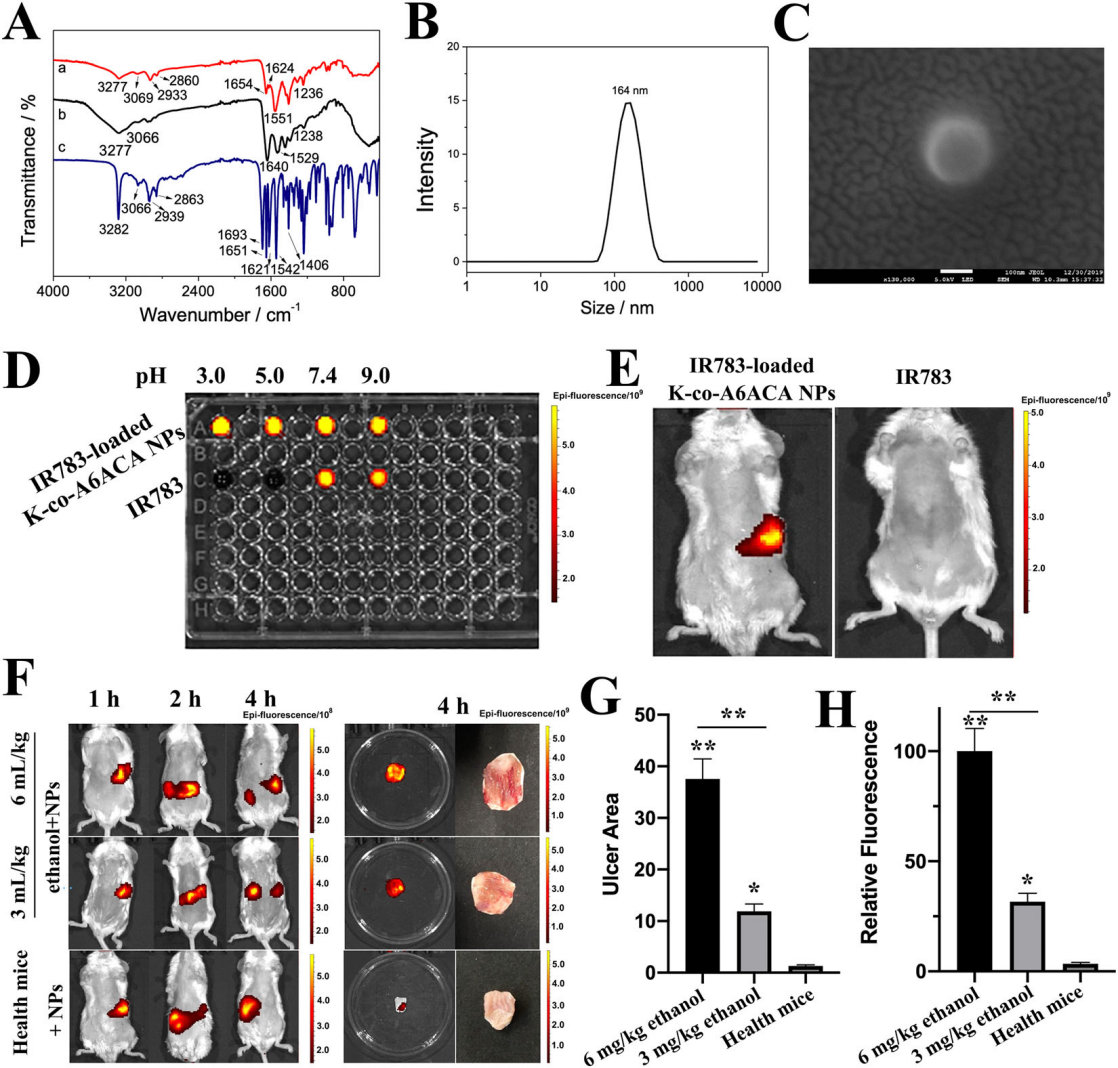
**A** FTIR spectrum of (a) keratin-co-A6ACA, (b) Keratin and (c) A6ACA. **B** The particle size distribution and SEM image of K-co-A6CAC NPs. **C** The in vitro fluorescence of IR783 and IR783-loaded NPs under different pH conditions. **D** The in vivo fluorescent signals of IR783 and IR783-loaded K-co-A6CAC NPs in healthy mice stomach. **E** The in vivo fluorescent signals, **F** ulcer areas, and **G** relative fluorescent intensity of IR783-loaded NPs in ethanol-induced mice with different gastric ulcer and health mice

The intensity of CO–NH bonds at 1551 and 1236 cm^−1^ significantly enhanced in the FTIR spectra of K-co-A6ACA compared to that in keratin and A6ACA (Fig. 1A), indicating the reaction occurred between the amino of keratin and the carboxyl of A6ACA. In addition, IR783-loaded K-co-A6ACA NPs in the shape of sphere with approximately 164 nm was observed (Fig. 1B, C).

The fluorescence from IR783 is quenched in acidic conditions (pH 3.0 and pH 5.0), and clear fluorescent signals can be observed in neutral (pH 7.4) and basic conditions (pH 9.0). While, IR783 kept its fluorescent signals in acidic condition after entrapped into NPs, demonstrating K-co-A6ACA could protect the IR783 from acidic solution (Fig. 1D). Furthermore, the in vivo fluorescence of IR783 and IR783-loaded NPs were detected in the health mice (Fig. 1E), and the same phenomenon was observed. The fluorescent signal of K-co-A6ACA NPs can be clearly seen, but the fluorescence from IR783 was quenched in the stomach. Next, the IR783-loaded NPs were fed into ethanol-induced mice with different ulcer and health mice, respectively (Fig. 1F). Stronger fluorescent signal can be observed within 4 h in case of larger ulcer formed in mice stomach (Fig. 1G), and the fluorescent intensity decreased ~68.33% when the gastric ulcer decreased from 37.55 ± 3.88 mm^2^ to 11.89 ± 1.43 mm^2^. While, few fluorescent signals were found in the health stomach. Furthermore, most of NPs retained in stomach with larger ulcer within 4 h, and a large number of NPs have been transported into the intestine with small gastric ulcer. While, most of NPs stayed in health mice intestine at the same time, suggesting NPs could retain in ulcer stomach for a long time due to its ulcer adhesive property, and the fluorescent intensity can be changed according to the area of gastric ulcer.

**Fig. 2:**
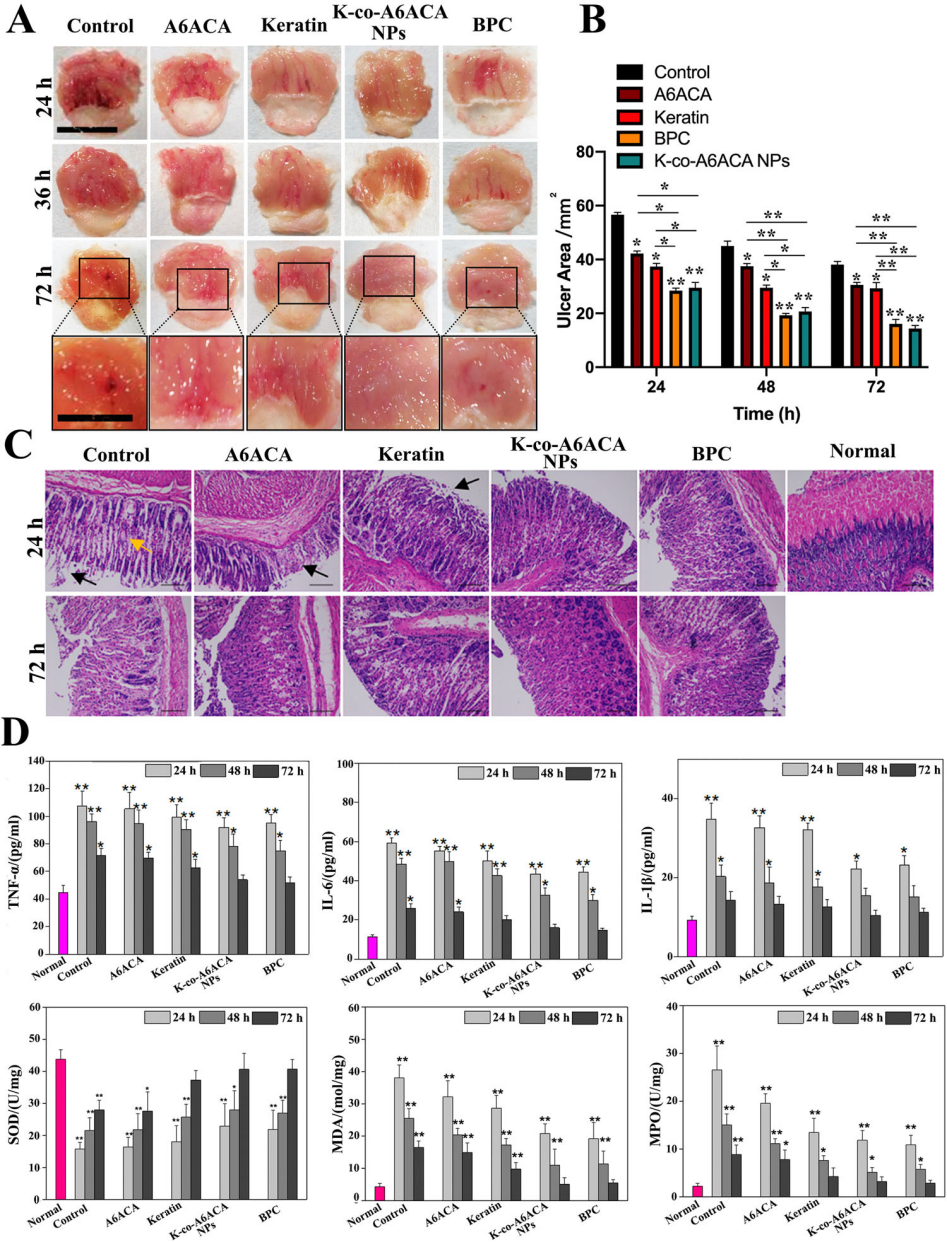
The therapeutic effect of K-co-A6CAC NPs on ethanol-induced gastric ulcer. **A** Macroscopic photograph and **B** ulcer areas of stomachs for ethanol-induced ulcer mice treated with or without A6CAC, keratin, BPC, and NPs at different times. **C** Histopathology of the stomach tissue (Magnification: ×200, yellow arrow: edema, and black arrows: loss of epithelial layer), **D** the content of TNF-α, IL-6, and IL-1β in mouse serum, and the content of SOD, MDA, and MPO in mice stomach after different treatments. *P < 0.05, **P < 0.01, compared with the control group

In addition, the therapeutic effects of NPs, keratin, A6ACA and BPC on gastric ulcer healing were investigated. The ethanol-induced gastric ulcer including hemorrhage and edema have been found (Fig. 2A). The obvious ulcer can be observed in the control group at 72 h, and the hemorrhage and edema alleviated after the keratin and A6ACA treatments. Furthermore, the ulcer almost healed completely after the NPs treatment at 72 h, while, there was still a certain degree of ulcer in BPC group. Furthermore, the gastric ulcer areas after different treatments within 72 h were measured (Fig. 2B). A6ACA, keratin, BPC, and NPs significantly reduced the ulcer area compared to control group, and a higher ulcer healing speed has been observed in the BPC and NPs groups than that in other groups. No significant difference in ulcer area between the BPC and NPs groups, but more serious symptoms including hemorrhage and edema have been observed in BPC therapy compared to that in NPs therapy.

The histopathology was assessed via H&E straining of the stomach tissue at 24 and 72 h (Fig. 2C). The edema (yellow arrow) and loss of epithelial layer (black arrows) have been found in control group. The histopathological changes induced by ethanol slightly relieved after A6ACA and keratin treatments at 72 h, and a small amount of necrosis can be seen on the surface of gastric mucosa in BPC group. While, the injured mucosal layer is completely recovery and same with the healthy stomach after NPs treatment. Furthermore, the ethanol feeding also caused the increase in TNF-α, IL-1β and IL-6 levels in serum and the content of MDA and MPO in stomach (Fig. 2D), and induced the decrease of SOD content. The K-co-A6ACA NPs could adjust them to normal levels at the fastest speed among the different treatments.

## Conclusions

In conclusion, we successfully fabricated the IR783-loaded K-co-A6ACA NPs for ethanol-induced gastric ulcer diagnosis and repair in this study, which could protect the IR783 dye and gastric epithelial cells from gastric juice, and enhance the ulcer healing based on the ulcer targeting and wound regeneration effects of keratin, demonstrating that it is a promising alternative for the diagnosis and treatment of gastric ulcer.
